# Origin of the 6/5/6/5 Tetracyclic Cyclopiazonic Acids

**DOI:** 10.3390/md22020074

**Published:** 2024-01-31

**Authors:** Wenyuan Zhang, Xuejian Jiang, Minjun Wang, Zhizhen Zhang, Nan Wang

**Affiliations:** 1 Ocean College, Zhejiang University, Zhoushan 316021, China; 11734051@zju.edu.cn (W.Z.); xuejian_jiang@zju.edu.cn (X.J.); 22034173@zju.edu.cn (M.W.); zzhang88@zju.edu.cn (Z.Z.); 2 Hainan Institute of Zhejiang University, Sanya 572025, China

**Keywords:** cyclopiazonic acid, indole alkaloids, sponge-associated fungus, tetramic acid, biosynthesis

## Abstract

The natural product α-cyclopiazonic acid (α-CPA) is a very potent Ca^2+^-ATPase inhibitor. The CPA family of compounds comprise over 80 chemical entities with at least five distinct skeletons. While α-CPA features a canonical 6/5/6/5/5 skeleton, the 6/5/6/5 skeleton is the most prevalent among the CPA family. However, the origin of the unique tetracyclic skeleton remains unknown. The 6/5/6/5-type CPAs may derive from a precursor of acetoacetyl-l-tryptophan (AATrp) generated from a hypothetic thioesterase-like pathway. Alternatively, cleavage of the tetramic acid ring would also result in the formation of the 6/5/6/5 scaffold. *Aspergillus oryzae* HMP-F28 is a marine sponge-associated filamentous fungus known to produce CPAs that act as primary neurotoxins. To elucidate the origin of this subfamily of CPAs, we performed homologous recombination and genetic engineering experiments on strain HMP-F28. Our results are supportive of the ring cleavage pathway through which the tetracyclic 6/5/6/5-type CPAs are generated from 6/5/6/5/5-type pentacyclic CPAs.

## 1. Introduction

The natural product α-cyclopiazonic acid (α-CPA) is a prenylated indole tetramic acid alkaloid with a characteristic 6/5/6/5/5 pentacyclic skeleton. It was first isolated from *Penicillium cyclopium* Westling in 1968 and was widely recognized as a neurotoxin that inhibits the action of sarco/endoplasmic reticulum Ca^2+^-ATPase (SERCA) at nanomolar concentrations [1,2]. It is widely used as a tool in pharmacological research to study the function of intracellular calcium signaling pathways [3,4]. In the food industry, α-CPA and its derivatives are indicators of fungal contamination [5,6]. About 80 CPA homologues have been reported from *Aspergillus*, *Penicillium*, *Chrysosporium*, and *Amycolatopsis* species [7,8,9]. An increasing number of new CPA derivatives with versatile bioactivities such as antitumor, anti-oxidation, anti-bacterial, and anti-TMV (tobacco mosaic virus) have been identified from fungal isolates of marine origins [10,11,12,13]. 

**Figure 1:**
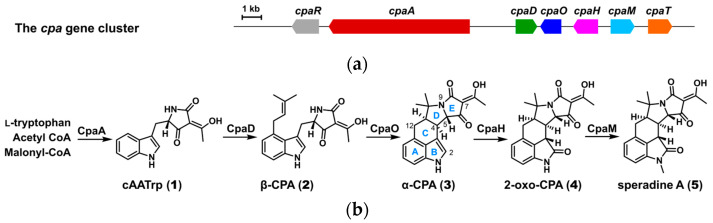
The biosynthetic gene cluster *cpa* (**a**) and CPA biosynthetic pathway (**b**) identified in the marine sponge-associated fungus *Aspergillus oryzae* HMP-F28. CPA: cyclopiazonic acid; cAATrp: *cyclo*-acetoacetyl-l-tryptophan; CpaR: transcription factor; CpaA: hybrid PKS–NRPS; CpaD: tryptophan dimethylallyltransferase; CpaO: monoamine oxidase; CpaH: cytochrome P450 monooxygenase; CpaM: *N*-methyltransferase; CpaT: MFS (major facilitator superfamily) transporter.

The known biosynthetic gene clusters (BGCs) of CPA involve either three (*cpaA/D/O*) or five (*cpaA/D/O/H/M*) biosynthetic genes to produce α-CPA and speradine A, respectively. A regulatory gene (*cpaR*) and a transporter gene (*cpaT*) are usually associated with these biosynthetic genes (Figure 1a) [14,15,16,17,18,19,20]. CpaA, a hybrid polyketide synthase–nonribosomal peptide synthetase (PKS–NRPS, also known as CpaS), generates the release product cAATrp (**1**, *cyclo*-acetoacetyl-l-tryptophan) from one molecule of l-tryptophan, acetyl CoA, and malonyl CoA. The unique tetramic acid (E ring) of cAATrp is formed via Dieckmann condensation catalyzed by the R* domain of CpaA during the non-reductive release of the PKS–NRPS product. A dimethylallyltransferase (CpaD) then adds a prenyl group at C4 to produce β-cyclopiazonic acid (β-CPA, **2**). CpaO, an FAD-dependent monoamine oxidase, is involved in the intramolecular cyclization of β-CPA to form the pentacyclic α-CPA (**3**) [21,22]. Some *cpa* gene clusters have two more tailoring genes encoding a cytochrome P450 monooxygenase (CpaH) and an *N*-methyl transferase (CpaM), respectively. They sequentially convert α-CPA to 2-oxo-CPA (**4**) and speradine A (**5**) (Figure 1b) [19,20].

**Scheme 1:**
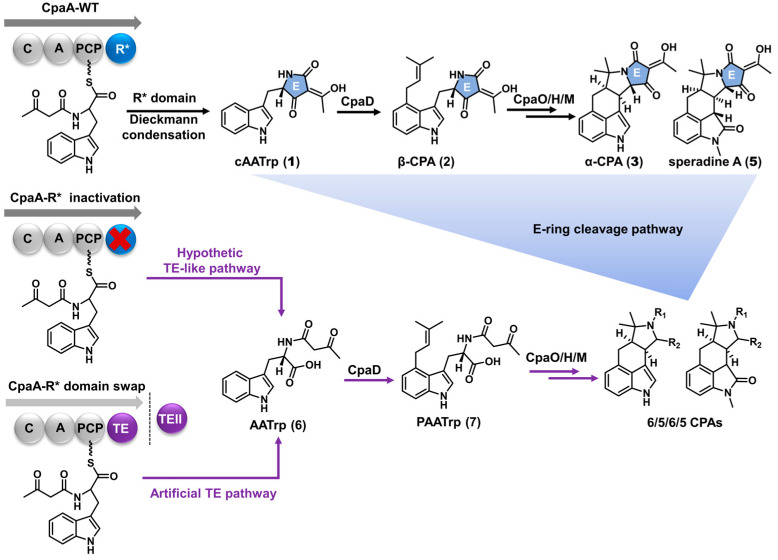
The established biosynthetic pathway (black arrows) generates cAATrp as the first offline precursor via Dieckmann reaction catalyzed by the nonreductive R* domain of CpaA (blue circle). The hypothetic TE-like pathway releases AATrp for downstream processing by the CPA biosynthetic machinery (purple arrows). The hypothetic retro-Dieckmann pathway produces the 6/5/6/5 skeleton directly from 6/5/6/5/5-type CPAs by E-ring cleavage (blue triangle). The R* domain was engineered to either enhance (cpaA-R* inactivation) the hypothetic TE-like pathway or introduce artificial TE mechanisms (TE swap and TEII introduction, purple circles).

According to the established CPA biosynthetic pathway, α-CPA and/or speradine A are expected to be the final products of the *cpa* gene clusters. Both of them possess a characteristic 6/5/6/5/5 pentacyclic skeleton. However, it is fascinating to note that the reported CPAs exhibit at least five distinct ring systems (Figure S1). Among them, CPAs with a 6/5/6/5 tetracyclic skeleton are the most commonly discovered congeners (>25 entities). The 6/5/6/5-type CPAs are devoid of the characteristic tetramic acid ring (E ring). Such structural variation may derive from two hypothetic pathways: the TE-like (thioesterase-like) pathway involving a hydrolytic release of the CpaA product, namely acetoacetyl-l-tryptophan (AATrp) instead of cAATrp, and the retro-Dieckmann pathway involving E-ring cleavage after the 6/5/6/5/5 skeleton formation (Scheme 1). The TE-like pathway requires an alternative release mechanism other than the non-reductive cyclization release mediated by CpaA-R*. It is possibly catalyzed by a discrete, *trans*-acting type II thioesterase (TEII), the product of which (AATrp) can be further processed by CpaD and CpaO to elaborate the tetracyclic scaffold. Apparently, the hypothetic TE-like pathway functions in a competitive manner with CpaA-R*. The retro-Dieckmann pathway, on the other hand, is either a spontaneous or an enzymatic ring-opening process. In this study, we used a marine sponge-associated fungus known as a CPA producer [23], *A. oryzae* HMP-F28, to test our hypothesis through *cpa* BGC engineering and feeding experiments. Our results are strongly supportive of the proposed retro-Dieckmann pathway, offering a biogenic interpretation of the widely-reported 6/5/6/5 subfamily of CPAs. 

## 2. Results and Discussion

### 2.1. The 6/5/6/5 Skeleton Is Not Derived from the TE-like Pathway

**Figure 2:**
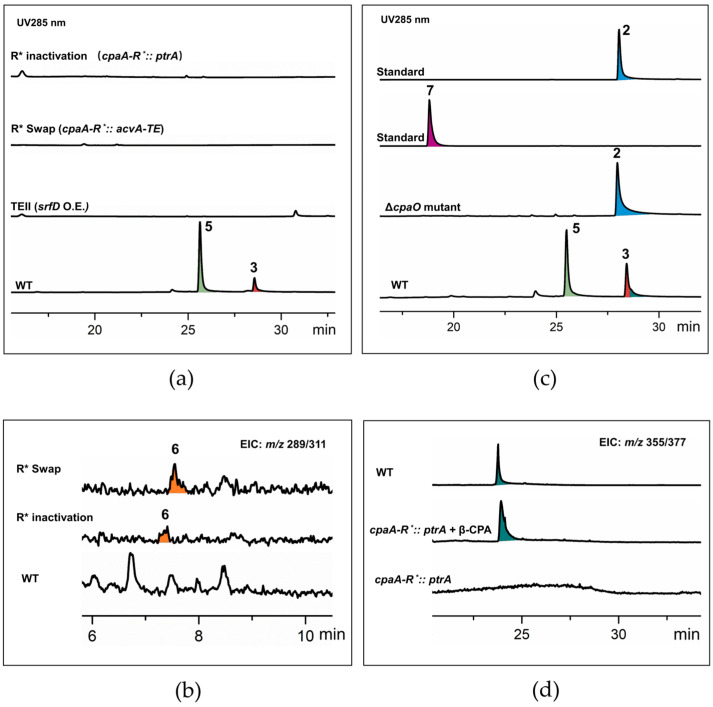
HPLC profile analysis (**a**) and LC-TOFMS detection of AATrp (**b**) in the R*-domain-inactivated (cpaA-R*::ptrA) and domain-swapped (cpaA-R*::acvA-TE) mutants in comparison with the WT strain. E-ring cleavage was not observed in β-CPA producing strain Δ*cpaO* (**c**), but when it was converted in to α-CPA and speradine A by the R*-domain knockout mutant, the 6/5/6/5-type CPAs were detected at *m*/*z* 355.1652 [M + H]^+^ and *m*/*z* 377.1472 [M + Na]^+^ (**d**).

To test the presence of the proposed TE-like pathway, we inactivated the cpaA-R* gene to abolish the R*-domain mediated Dieckmann condensation forming the tetramic acid ring while releasing the PKS–NRPS product (Scheme 1). By eliminating the competition with the hypothetic TE-like pathway, we expected to see a significant increase in the production of 6/5/6/5 products. The R* domain was knocked out by inserting the *ptrA* gene to generate the cpaA-R*::ptrA mutant. When compared to the wild-type (WT) strain, the production of pentacyclic α-CPA (**3**) and speradine A (**5**) in the mutant strain was eliminated. Further, the desired 6/5/6/5 tetracyclic products were not detected in the R*-domain disrupted mutant (Figure 2a). This result suggests that there is not such a competitive hydrolytic release mechanism that generates the intermediate AATrp with an uncyclized polyketide chain (acetoacetyl chain).

### 2.2. AATrp Is a Shunt Metabolite

To further verify if AATrp is a valid intermediate being processed by the CPA biosynthetic machinery to produce the 6/5/6/5 products, we genetically deleted the cpaA-R* domain in the WT strain and complemented it with the *acvA-TE* gene from *A. nidulans*. The *acvA-TE* gene encodes a TE domain [24]. The resulting cpaA-R*::acvA-TE mutant with an artificial TE mechanism was expected to release AATrp as precursor for downstream biosynthetic processes (Scheme 1). LC-TOFMS analysis showed that the domain-swapped strain was unable to produce the 6/5/6/5-type tetracyclic CPAs (Figure 2a). Nonetheless, AATrp was observed in the cpaA-R*::ptrA and the cpaA-R*::acvA-TE mutants, albeit in very low amount (Figure 2b). A thorough analysis of the extracted ion chromatogram (EIC) did not reveal any downstream products generated by CpaD or/and CpaO (Table S4). That the titer of AATrp in the domain-swapped strain appeared higher than the domain-inactivated strain assumes successful TE-mediated release by AcvA-TE (Figure 2b). Accumulation of AATrp and absence of any downstream products demonstrated that AATrp is not used for prenylation by CpaD, and cannot be incorporated into the CPA biosynthetic pathway (Scheme 1). We note that although AATrp may be generated via nonspecific hydrolysis, and from our genetically modified fugal strains (cpaA-R*::acvA-TE mutants), it still cannot be utilized as a substrate by CpaD. This further verified that the production of 6/5/6/5-type CPAs through the proposed TE-like pathway cannot occur.

### 2.3. The E-Ring Cleavage Occurs after 6/5/6/5/5 Skeleton Formation

**Scheme 2:**
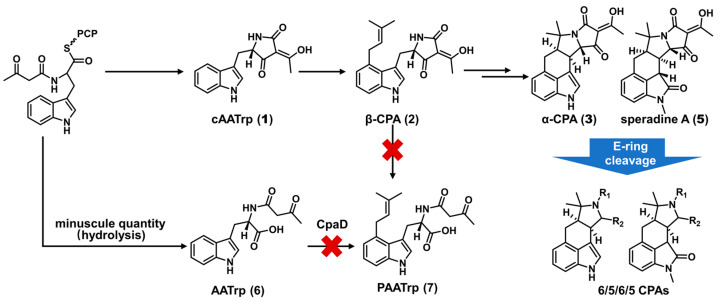
The proposed origins of the 6/5/6/5-type CPAs.

The above results imply that the 6/5/6/5 skeleton may derive from the E-ring (tetramic acid) cleavage pathway. The cleavage chemistry should take place after β-CPA formation, because CpaD does not recognize the ring-opening product (AATrp) of cAATrp for prenylation to generate PAATrp (**7**, prenylated AATrp, Scheme 1, Table S5). To detect the timing of E-ring cleavage, we conducted metabolic profiling experiments in the WT, Δ*cpaO* and the R*-domain inactivated mutant strains. As expected, the *cpaO*-inactivated strain accumulated a single metabolite identified as β-CPA (**2**, Figure 2c, Table S5). After prolonged fermentation (9 days), β-CPA remained pretty stable, and no PAATrp or PAATrp derivatives were observed (Figure 2c). Extensive high-resolution EIC analysis (Table S4) were carried out to monitor the production of 6/5/6/5 derivatives. In the WT strain cultured for 16 days, ion peaks at *m/z* 355.1652 [M+H]^+^ and *m/z* 377.1472 [M + Na]^+^ were observed while producing *cpa* products α-CPA (**3**) and speradine A (**5**). Both ions were also found as the major CPA-derived molecular adducts in the R* domain disrupted mutant supplemented with β-CPA (Figure 2d). Shown in Table S4, the ion pairs are in agreement with the known tetracyclic products speradine I [25] (note that there is an earlier report used “speradine I” for another CPA congener [26]) and a hypothetic ring-opening derivative named as *seco*-α-CPA. The two compounds show obvious origins of speradine A and α-CPA, respectively. Also, other 6/5/6/5-type species at *m/z* 371.1553/393.1425 and 385.1774/407.1575 were also observed (Table S4). Listed in Table S4 are the hypothetic E-ring cleavage products subjected to high-resolution EIC analysis. These findings support the hypothesis that the 6/5/6/5 skeleton is formed after α-CPA formation. Accordingly, we propose that the 6/5/6/5-type tetracyclic CPAs are derived from the 6/5/6/5/5-type CPAs. Successive decarboxylation and oxidation lead to the 5-oxo 6/5/6/5-type skeleton which is the predominant form of known CPAs (Scheme 2). The cleavage chemistry of the E ring is therefore assumed to be an intriguing retro-Dieckmann reaction.

All the genes in the *cpa* gene cluster have been at least partially characterized [14,15,16,18,19,20]. None of them are potentially involved in the retro-Dieckmann reaction. Since the cpaA-R*-knockout mutant still retained the ability to generate tetracyclic products when feeding with β-CPAs (chemical complementation), it is certainly not a reversed reaction promoted by the R* domain. The majority of the tetracyclic CPAs were isolated from cultures of rice-based solid media, with the fermentation period typically exceeding 30 days [27]. During the over-prolonged culture periods, it is not surprising that the ring cleavage reaction may occur either spontaneously or as a result of a nonspecific enzyme encoded by a gene outside of the *cpa* gene cluster. In the latter case, it would be particularly intriguing to identify the unprecedent enzyme catalyzing the retro-Dieckmann reaction of tetramic acid.

## 3. Materials and Methods

### 3.1. General Experimental Procedures

LC-MS was performed on an Agilent 1260 Infinity LC coupled to a 6230 TOF (HRESI) equipped with an YMC-ODS-A C18 column (250 × 4.6 mm, 5 µm) (YMC Co., Ltd., Kyoto, Japan) with a constant temperature of 35 °C. Analytic and semi-preparative HPLC was performed on a SHIMAZU LC-20 equipped with a DAD detector using a YMC-ODS-A C18 column (250 × 4.6 mm, 5 µm) and a YMC-ODS-A C18 column (250 × 10.0 mm, 7 µm), respectively. NMR spectra were recorded in CDCl_3_ and CD_3_OD on a JEOL JNM-ECZ600R/S1 spectrometer (600 MHz) (JEOL, Tokyo, Japan). HRESIMS spectra were measured on an Agilent 6230 TOF mass spectrometer (Agilent Technologies, Palo Alto, CA, USA).

### 3.2. Strains and Reagents

*A. oryzae* HMP-F28 was isolated from a marine sponge (*Hymeniacidon perleve*) collected from the Bohai Sea at Heishi Reef Bay (38°51′52.4′′ N 121°33′05.0′′ E) in Dalian, China. HMP-F28 was cultured on Czapek–Dox (CD) medium prepared with artificial sea water containing 2.35% NaCl, 1.078% MgCl_2_, 0.147% CaCl_2_, 0.4% Na_2_SO_4_, 0.068% KCl, 0.0196% NaHCO_3_, 0.0026% borax, 0.00003% EDTA, and 0.003% Na_2_SiO_3_. Mutants were cultivated on CD medium containing 0.25 µg/mL pyrithiamine as the selection marker. Other strains and plasmids used in this study are listed in Supplementary Table S1. PCR primers described in Supplementary Table S2 were synthesized by Sunya (Hangzhou, China). Enzymes for cloning and gene editing were purchased from TAKARA (Takara Bio Inc., Beijing, China) and NEB (NEB, Beijing, China) and used following the protocols provided by the manufacturer. DNA gel extraction and plasmid preparation kits were purchased from Sangon (Shanghai, China). DNA sequencing was conducted by Personalbio (Shanghai, China) and Sunya (Hangzhou, China). *Escherichia coli* DH5α was used for general cloning and sub-cloning. Regular chemicals and biochemical and media components were purchased from regular commercial sources, including Sinopharm, Oxoid, and Macklin.

### 3.3. HPLC and LC-TOFMS Analysis

The following LC-MS or HPLC analytical method was used throughout this study unless otherwise stated: YMC C18 column (4.6 mm × 250 mm, 5 µm); mobile phase A: H_2_O containing 0.1% (*v/v*) formic acid; mobile phase B: acetonitrile. Elution gradient (*v/v*): 0–8 min, 37% B; 8–15 min, 37–63% B; 15–25 min, 63–100% B; 25–33 min, 100% B. Flow rate: 0.6 mL/min; detection: DAD/260 nm or ESITOFMS. Taking advantage of the high production background of CPAs in HMP-F28, cultures of WT and mutants were centrifuged at 13,000 × *g* for 20 min. The supernatant was then transferred to autosampler vials for LC-MS or HPLC analysis without further purification.

### 3.4. Engineering of the cpaA-R* Domain

The cpaA-R* domain was disrupted by homologous recombination with a DNA fragment comprising two 1 kb homology arms flanking the *ptrA* gene. The homology arms were amplified from the genomic DNA of wild-type *A. oryzae* HMP-F28. The resistance gene *ptrA* was amplified from the pPTR Ⅱ plasmid and was used as the selection marker. The two amplicons and *ptrA* were assembled using overlap PCR (Figure S2a). For the CpaA-R* domain swap, several TE domains were summarized in Table S3. The *acvA-TE* gene was synthesized by Genewiz (Suzhou, China), which releases a linear tripeptide of ACV (l-δ-(α-aminoadipoyl)-l-cysteinyl-d-valine) in the *acv* biosynthetic gene cluster [24,28]. The vector for the domain swap was constructed using overlap PCR assembling the homology arms, *ptrA* fragment, and the *acvA-TE* fragments (Figure S3a). Similarly, for overexpression of the *srfD* gene encoding a TEII from *Bacillus subtilis* [29], the linear DNA for homologous recombination was assembled with the *srfD*, *pamyB*, and *tamyB* fragments shown in Figure S4a. Transformation was performed using the PEG-mediated transformation of protoplasts [30]. In brief, gene targeting DNA were transformed into protoplasts and poured into the double-layer CD plate containing 0.25 µg/mL pyrithiamine, with or without combined selection markers including 1.0–1.5 mg/mL 5-fluoroorotic acid, 0.5% uridine and 0.2% uracil. The plate was incubated at 28 ℃ for transformants to appear. The genotypes of the mutants were verified by diagnostic PCRs using the appropriate primers listed in Table S2. Transformants II33 and II41 were identified as the correct cpaA-R*::ptrA mutants, and ss6 as the correct cpaA-R*::acvA-TE mutant (Figures S2b and S3b). For the *srfD* expressing mutants, both growth phenotype analysis and diagnostic PCR were used for transformant screening (Figure S4b,c). The metabolic phenotypes of each mutant were profiled by LC-MS and/or HPLC analysis.

### 3.5. Biotransformation Assays

Strains were cultured in 250 mL flasks containing 100 mL of CD medium. Either β-CPA or α-CPA was added to the fungal cultures on day 0 to final concentrations of 100 mg/mL and 0.1 mg/mL, respectively. After cultivating for 7 days, 9 days, and 16 days at 200 rpm 28 °C on a rotary shaker, the supernatant of the cultures was harvested by centrifuge at 13,000 × *g* and subjected to LC-MS or HPLC analysis.

### 3.6. Isolation and Purification

Compounds *α*-CPA and β-CPA were isolated from large-scale fermentation of the Δ*cpaH* and Δ*cpaO* mutants, respectively. Briefly, eighty flasks each containing 200 mL CD medium were cultured at 28 °C with shaking at 200 rpm for 7 days. The cultures were harvested and filtred through gauze. The resulting filtrate was then subjected to XAD-16 resin (30 g/L) adsorption. After washing the resin with water to remove the impurities, the crude extracts were obtained by elution of the resin with methanol (3 column volumes). Compounds α-CPA and β-CPA were purifed using HPLC over a semi-preparative ODS column (Section 3.1) eluted isocratically with 70% methanol (*v/v*) at 3 mL/min.

### 3.7. Synthesis of AATrp and PAATrp

AATrp was synthesized as described elsewhere with slight modifications [31]. In brief, l-tryptophan and K_2_CO_3_ were added to a round bottom flask containing 10 mL pure H_2_O to the final concentrations of 10 mM and 8 mM, respectively. The reaction mixture was heated to boiling. Upon reflux, 2, 2, 6-trimethyl-4*H*-1, 3-dioxin-4-one (TMD, 20 mM) was added and maintained the reflux with stirring for 2.5 h. The cooled reaction mixture was acidified with aqueous 6 M HCl (pH 1) and extracted with EtOAc (1:1, *v/v*) 3 times. The organic phase was collected and evaporated to dryness under vacuum (Figure S5).

PAATrp was obtained similarly with TMD and 4-dimethylallyl tryptophan (4-DMAT) as reactants. Compound 4-DMAT was prepared by biotransformation with a *fgaPT2*-, *phoN*-, *ipK*-overexpressing *E. coli* Rosetta (DE3) [32]. The *fgaPT2* gene from *Aspergillus fumigatus* encodes a C4-prenyltransferase using l-Trp as substrate (Figure S5) [33]. The *fgaPT2* was synthesized and inserted into a pET28a vector, which was transformed into *E. coli* for FgaPT2 production and subsequent prenylation to produce 4-DMAT (Figure S5).

## 4. Conclusions

CPAs are a group of mycotoxins produced from both terrestrial- and marine-derived fungi that are in the process of being understood. However, according to known *cpa* biosynthetic machineries, only cAATrp, β-/α-CPA, 2-oxo-CPA and speradine A are genuine natural products generated by the CPA biosynthetic gene clusters as either intermediate or final products. As a result, it is hard to rationalize the biogenesis of the discovered chemical diversity of CPAs. In this study, we probed the origin of the representative 6/5/6/5 skeleton of CPAs. Our results showed that the biosynthetic machinery of CPA is specific for its intermediates, especially for the first offline product cAATrp. Our R* domain engineering provides robust evidence that the 6/5/6/5 skeleton of CPAs are not derived from the hypothetic TE-like pathway. Further in vivo biotransformation showed that the 6/5/6/5-type CPAs were formed after the 6/5/6/5/5 scaffold was synthesized by the CPA biosynthetic machinery (Scheme 2).

## Data Availability

The original data presented in the study are included in the article/Supplementary Material; further inquiries can be directed to the corresponding author.

## Supplementary Materials

The supporting information on molecular cloning, gene editing, HRESITOFMS analysis, NMR data assignments, and NMR spectra can be downloaded at: https://www.mdpi.com/article/10.3390/md22020074/s1. Refs. [24,32,34,35,36,37].
