# Supplementary material for: Origin of the 6/5/6/5 Tetracyclic Cyclopiazonic Acids
Source: Mar Drugs. 2024 Jan 31;22(2):74. doi: 10.3390/md22020074 (PMC10890092; doi:10.3390/md22020074)
Supplement: Supplementary file 1 [file marinedrugs-22-00074-s001.zip › marinedrugs-2852486-supplementary_revised.pdf]

Supplementary Materials

Origin of the 6/5/6/5 Tetracyclic Cyclopiazonic Acids

Wenyuan Zhang [1], Xuejian Jiang [1], Minjun Wang [1], Zhizhen Zhang [1] and Nan Wang [1,2,*]

[1] Ocean College, Zhejiang University, Zhoushan 316021, China; n_wang@zju.edu.cn
[2] Hainan Institute of Zhejiang University, Sanya 572025, China; n_wang@zju.edu.cn
* Correspondence: n_wang@zju.edu.cn (N.W.)

Content

Table S1. Strains, plasmids and mutants in this study 2
Table S2. Primers used in this study 2
Table S3. TE domains of different origins 3
Table S4. Hypothetic E-ring opening compounds subjected for EIC analysis 4
Table S5. NMR assignments for β-CPA, α-CPA and PAATrp (^{1}H, 600 MHz, ^{13}C 150 MHz) 10
Figure S1. The reported skeletons of CPA-type compounds 6
Figure S2. Construction (a) and genotype verification (b) of *cpaA-R*::ptrA* mutants 7
Figure S3. Construction (a) and genotype verification (b) of the domain swapped mutants (*cpaA-R*::acvA-TE*) 7
Figure S4. Construction (a), screening (b) and verification (c) of the TEII expressing mutant by introduce *srfD* gene encoding a TEII to a *cpaA-R*::ptrA* mutant 8
Figure S5. Synthesis of AATrp and PAATrp by whole cell biotransformation and chemical reactions (a). The plasmids used for biosynthesis of DMAPP (dimethylallyl diphosphate) (pLD10001) and prenylation of L-tryptophan (pET28a-fgaPT2) in *E. coli* Rosetta (DE3) (b) 9
Figure S6. ^{1}H NMR spectrum of compound **2** (β-CPA) (600 MHz, $CDCl_3$)........11
Figure S7. ^{13}C NMR spectrum of compound **2** (β-CPA) (150 MHz, $CDCl_3$).......11
Figure S8. ^{1}H NMR spectrum of compound **3** (α-CPA) (600 MHz, $CDCl_3$)....... 12
Figure S9. ^{13}C NMR spectrum of compound **3** (α-CPA) (150 MHz, $CDCl_3$) 12
Figure S10. ^{1}H NMR spectrum of compound **7** (PAATrp) (600 MHz, $CDCl_3$)... 13
Figure S11. ^{13}C NMR spectrum of compound **7** (PAATrp) (150 MHz, $CDCl_3$).. 13
Figure S12. HMBC spectrum of compound **7** (PAATrp) (CD_3OD)........ 14
>acvA-TE 15
>fgaPT2 15

Table S1. Strains, plasmids and mutants in this study.

Strain / Plasmid	Genotype/Function	Source
A. oryzae HMP-F28	Wild type strain	In-house collection
cpaA-R::ptrA*	HMP-F28 derived CpaA-R* domain deletion mutant	This study
cpaA-R::acvA-TE*	HMP-F28 derived CpaA-R* domain swapped with AcvA-TE domain mutant	This study
E. coli DH5α	Host for general cloning	Takara
pUC 57-sgRNA	sgRNA carrier	GensScript
pUC 57-TE (AcvA)	TE (AcvA) domain carrier	GenScript
pUC 57-SrfD	TEII (SrfD) carrier	Genewiz
pPTR II	*ptrA* carrier	In-house collection
pLD10001	Expression of PhoN and IpK	This study
pET28a-fgaPT2	Expression of FgaPT2	This study

Table S2. Primers used in this study.

Primer	Nucleotide Sequence (5'-3')	Function
R*KO/Swap_up_F	AGAACTGTCCTGTGGTGCAGGACTCCTATGGTGTGACCGAGATTTC	PCR targeting for R* domain knock-out or swap
R*KO_up_R	agaatagcccgagatagagttgagTTATTGCGACTGGAGGGCAAATTCG	PCR targeting for R* domain knock-out
R*Swap_up_R	ctggcggcaataggaacaagatcggACAACCTGATCCTTGGCAATG	PCR targeting for R* domain swap
R*Swap_AcvA_F	agcattgccaaggatcaggttgtCCGATCTTGTTCCTATTGC	PCR targeting for R* domain swap
R*Swap_AcvA_R	agaatagcccgagatagagttgagCTAGAGAAATTTCTCGATTGTTGAACAAACGTG	PCR targeting for R* domain swap
R*KO_ptrA_F	atttgccctccagtcgcaataaCTCAACTCTATCTCGGGCTATTCTTTTGATTTATAAG	PCR targeting for R* domain knock-out
R*Swap_ptrA_F	ttcaacaatcgagaaatttctctagCTCAACTCTATCTCGGGCTATTCTTTTGATTTATAAGG	PCR targeting for R* domain swap
R*KO/Swap_ptrA_R	gaataagtggctcaataccctgAATAAGGGCGACACGGAAATGTTGAATAC	PCR targeting for R* domain knock-out or swap
R*KO_down_F	tcaacatttccgtgtcgcccttattCAGGGTATTGAGCCACTTATTCCGAGTTTC	PCR targeting for R* domain knock-out
R*Swap_down_F	tcaacatttccgtgtcgcccttattGGCAACAAGATACTCTTAACAC	PCR targeting for R* domain swap
R*KO/Swap_down_R	AGATCTCCATTGCTCAGCTTTCGGGTGCCTGG	PCR targeting for R* domain knock-out or swap
R*KO_ID_up-ptrA_F	CTGAATCACCATCAATGACAATCAACTG	PCR confirmation
R*KO_ID_up-ptrA_R	AATGAACTCGTTAGCTCGTAATCCAC	PCR confirmation
R*KO_ID_down-ptrA_F	CTGAGATAGAGAATTGTGTGGGATGAG	PCR confirmation
R*KO_ID_down-ptrA_R	TACCCTGAATCAGAACTGCATAAGC	PCR confirmation

R*Swap_ID_acvA-ptrA_F	ATCCCAACCAGAACCAGCCGATTTCC	PCR confirmation
R*Swap_ID_acvA-ptrA_R	GTGCCATGGTTCTCAGTGGTGTCAAGG	PCR confirmation
R*Swap_ID_up-acvA_F	GGTCAGATTACAAGGGGCGATC	PCR confirmation
R*Swap_ID_up-acvA_R	AACTTGTCGTTCACCCTAGTAGC	PCR confirmation
R*Swap_ID_ptrA-down_F	AGGAGGGGTTGAGTTAAATAGCATG	PCR confirmation
R*Swap_ID_ptrA-down_R	GATAACGCATTCTGAGATGTAAAACACAG	PCR confirmation
srfDExp_up_F	TATATGCTGCCACTGGTGGTTCTCCACTTG	PCR targeting for *srfD* expression
srfDExp_up_R	taaaatgatcaaaacaccatGTTGGCGATGGAGGGGTAG	PCR targeting for *srfD* expression
srfDExp_Pro_amyB_F	gctacccctccatcgccaacATGGTGTTTTGATCATTTTAAATTTTTATATG	PCR targeting for *srfD* expression
srfDExp_Pro_amyB_R	gatttaaagagctggaccatTGTGGGGTTTATTGTTCAGAGAAGG	PCR targeting for *srfD* expression
srfDExp_srfD_F	tctctgaacaataaaccccacaATGGTCCAGCTCTTTAAATC	PCR targeting for *srfD* expression
srfDExp_srfD_R	cttcacgagctactacagatcCTATAACAATTGGCCTGC	PCR targeting for *srfD* expression
srfDExp_Tmn_amyB_F	ctgcaggccaattgttatagGATCTGTAGTAGCTCGTGAAG	PCR targeting for *srfD* expression
srfDExp_Tmn_amyB_R	gagtacgtatccaccactacGGATCCTTTCCTATAATAGAC	PCR targeting for *srfD* expression
srfDExp_down_F	tctattataggaaaggatccGTAGTGGTGGATACGTACTCCTTTTATG	PCR targeting for *srfD* expression
srfDExp_down_R	GTTGCGGATCTTGCTGCTTGCGGAG	PCR targeting for *srfD* expression
pET28aFgaPT2N_His_F	GGAATTCCATATGATGAAGGCAGCCAATGCCTC	PCR targeting for *fgaPT2* expression with his tag
pET28aFgaPT2N_His_R	CCCAAGCTTTCAGTGGAGACCGGAATAATATACC	PCR targeting for *fgaPT2* expression with his tag

Table S3. TE domains of different origins.

NRPS/PKS	Structure	Source	Reference
AcvA	A-T-C-A-T-C-A-T-E-TE	*Aspergillus nidulans*	[24]
ApvA	A-T-TE	*Aspergillus terreus*	[34]
BtyA	A-T-TE	*Aspergillus terreus*	[34]
PngA	A-T-TE	*Aspergillus terreus*	[34]
PesM	A-T-C-A-T-C-E-A-T-C-TE	*Aspergillus fumigatus* AF293	[35]
SrfA-C	C-A-PCP-TE	*Bacillus subtilis*	[36]
TMC	KS-AT-KR-ACP-TE	*Streptomyces* sp. CK4412	[37]

Table S4. Hypothetic E-ring opening compounds subjected for EIC analysis.

No.	Structure	Calculated Mass	EIC Found
1	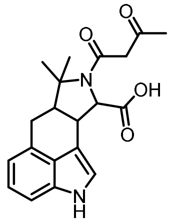 *seco*-α-CPA	$[M+H]^+$, *m/z* 355.1652, $[M+Na]^+$, *m/z* 377.1472	355.1647/377.1477 (WT, R_t 15~16 min); 355.1651/377.1455 (WT, R_t 19~20 min); 355.1656/377.1471 (WT, R_t 25~26 min)
2	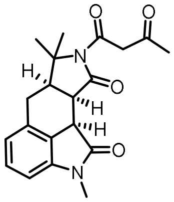 speradine I	$[M+H]^+$, *m/z* 355.1652, $[M+Na]^+$, *m/z* 377.1472	355.1647/377.1477 (WT, R_t 15~16 min); 355.1651/377.1455 (WT, R_t 19~20 min); 355.1656/377.1471 (WT, R_t 25~26 min)
3	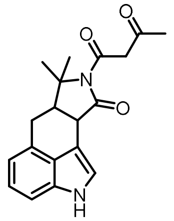 asperorydine F	$[M+H]^+$, *m/z* 325.1547, $[M+Na]^+$, *m/z* 347.1366	Not Detected
4	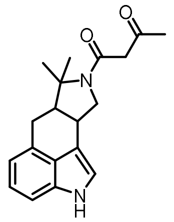	$[M+H]^+$, *m/z* 311.1754, $[M+Na]^+$, *m/z* 333.1573	Not Detected
5	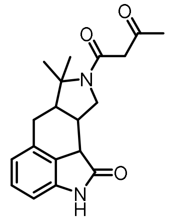	$[M+H]^+$, *m/z* 327.1703, $[M+Na]^+$, *m/z* 349.1523	Not Detected
6	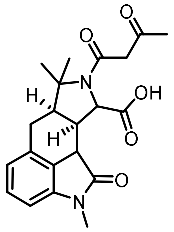	$[M+H]^+$, *m/z* 385.1758, $[M+Na]^+$, *m/z* 407.1577	385.1774/ 407.1575 (WT, R_t 12~13 min); 385.1769/407.1572 (*cpaA-R*::ptrA* + β-CPA, R_t 12~13 min)
7	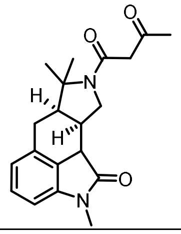	$[M+H]^+$, *m/z* 341.1860, $[M+Na]^+$, *m/z* 363.1679	Not Detected

8	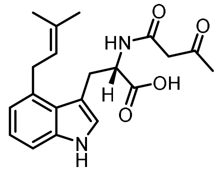 PAATrp	[M+H]$^+$, *m/z* 357.1809, [M+Na]$^+$, *m/z* 379.1628	357.1801/379.1622 (*cpaA-R*::ptrA* + PAATrp, R_t 18~19 min)
9	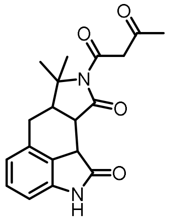	[M+H]$^+$, *m/z* 341.1496, [M+Na]$^+$, *m/z* 363.1315	Not Detected
10	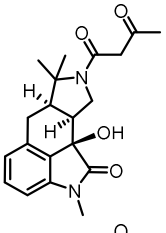	[M+H]$^+$, *m/z* 357.1809, [M+Na]$^+$, *m/z* 379.1628	Not Detected
11	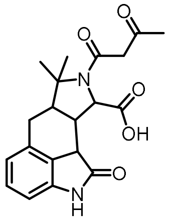	[M+H]$^+$, *m/z* 371.1601, [M+Na]$^+$, *m/z* 393.1421	371.1553/393.1373 (*cpaA-R*::ptrA* + α-CPA, R_t 15~16 min); 371.1606/393.1425 (*cpaA-R*::ptrA* + β-CPA, R_t 14~15 min);
12	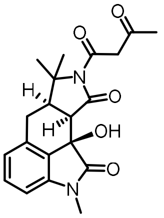 speradine C	[M+H]$^+$, *m/z* 371.1601, [M+Na]$^+$, *m/z* 393.1421	371.1553/393.1373 (*cpaA-R*::ptrA* + α-CPA, R_t 15~16 min); 371.1606/393.1425 (*cpaA-R*::ptrA* + β-CPA, R_t 14~15 min);
13	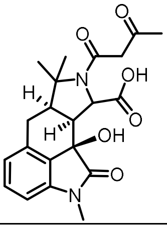	[M+H]$^+$, *m/z* 401.1707, [M+Na]$^+$, *m/z* 423,1527	Not Detected

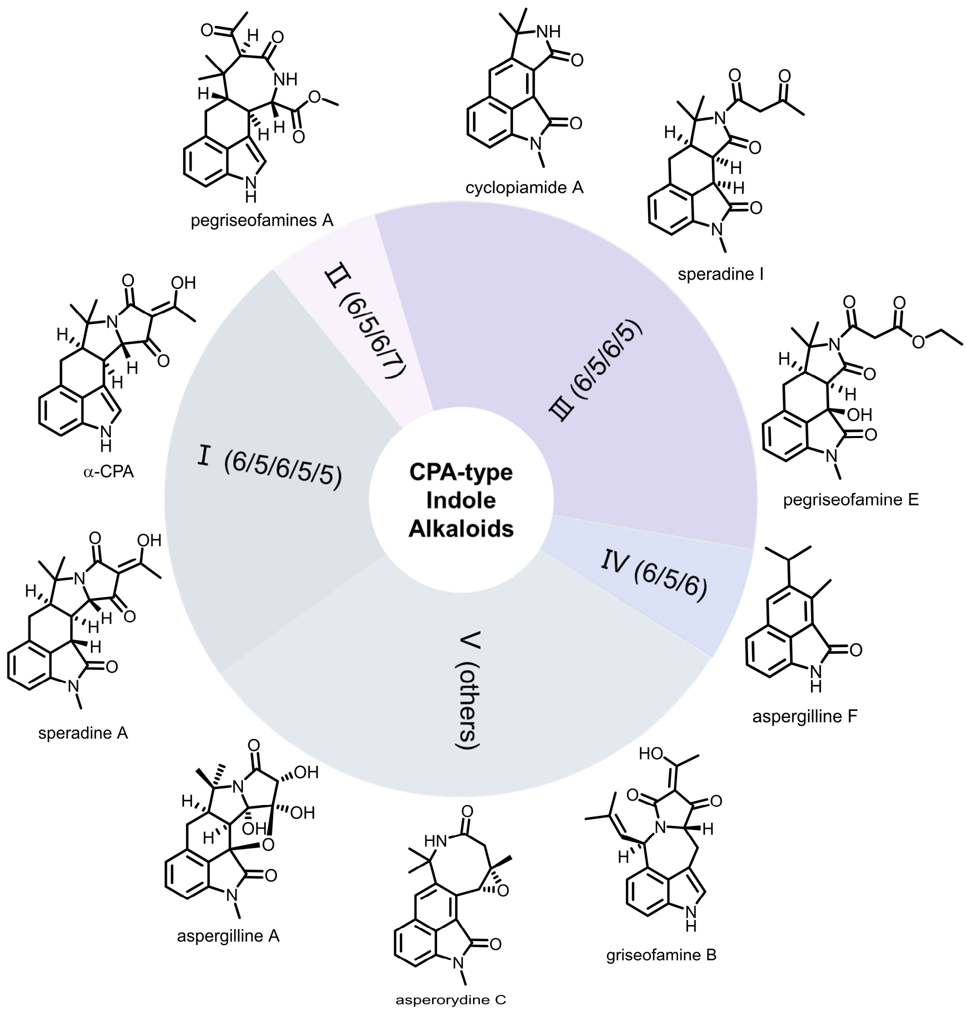

Figure S1. The reported skeletons of CPA-type compounds.

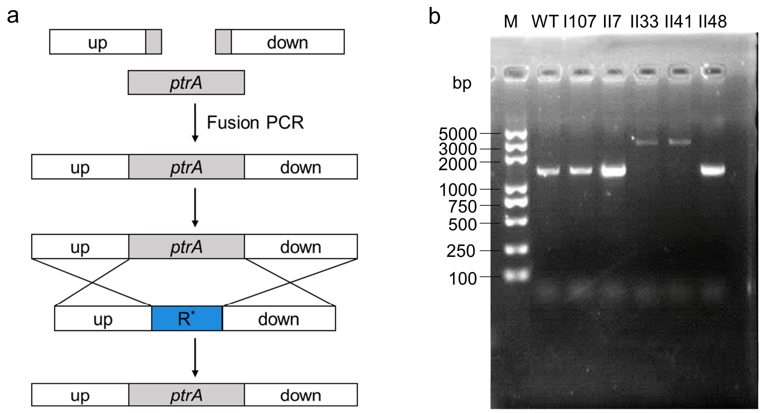

Figure S2. Construction (a) and genotype verification (b) of *cpaA-R*::ptrA* mutants. a. the diagram for *cpaA-R** gene knockout by homologous recombination, up and down: homology arm of *cpaA-R**, *ptrA*: pyrithiamine; b. agarose gel electrophoresis of *cpaA-R*::ptrA* mutants showed that II33 and II41 were identified to be the correct mutants, M: DNA marker.

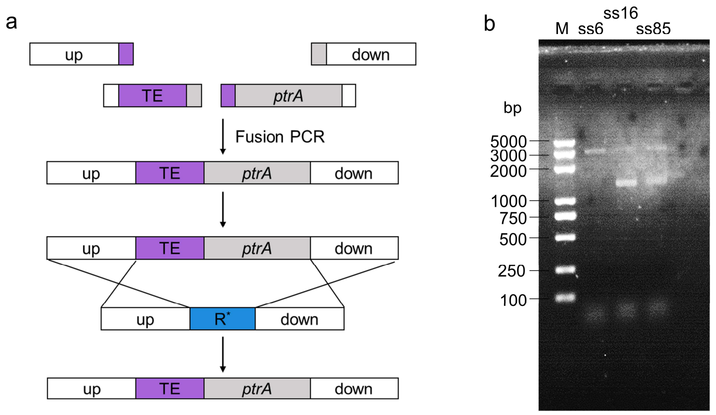

Figure S3. Construction (a) and genotype verification (b) of the domain swapped mutants (*cpaA-R*::acvA-TE*). a. the diagram showing replacement of *cpaA-R** with *acvA-TE* by homologous recombination, up and down: homology arm of *cpaA-R**, *ptrA*: pyrithiamine, TE: AcvA-TE domain; b. Transformant ss6 was identified as a correct mutant by agarose gel electrophoresis, M: DNA marker.

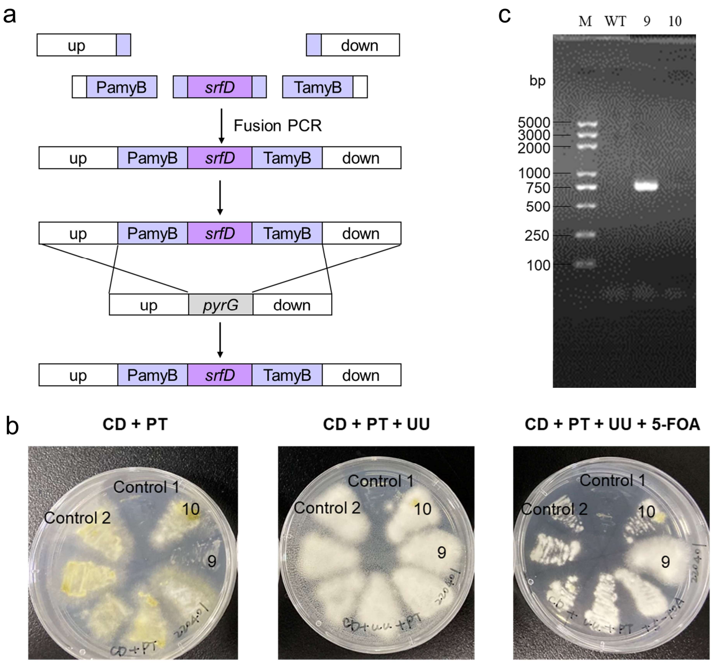

Strains	CD + PT	CD + PT + UU	CD + PT + UU + 5-FOA
Control 1:WT	x	x	x
Control 2: *cpaA-R*::ptrA* mutant	√	√	x
cpaA-R::ptrA*-OE srfD-9	x	√	√
cpaA-R::ptrA*-OE srfD-10	√	√	x

Figure S4. Construction (a), screening (b) and verification (c) of the TEII expressing mutant by introduce *srfD* gene encoding a TEII to a *cpaA-R*::ptrA* mutant. a. construction of the *srfD* overexpressing DNA fragment for homologous recombination, up and down: homology arm of *pyrG*; *pyrG*: orotidine-5'-phosphate decarboxylase, srfD: thioesterase, PamyB: amyB promoter, TamyB: amyB terminator; b. growth phenotype of *cpaA-R*::ptrA*-OE srfD mutants, WT strain (Control 1, wild type) couldn't growth on the CD+PT, CD+PT+UU and CD+PT+UU+5-FOA plates; the *cpaA-R*::ptrA* mutants (Control 2) with the *ptrA* marker grew on CD+PT and CD+PT+UU plates, but could not grow on CD+PT+UU+5-FOA plate; the correct *cpaA-R*::ptrA*-OE *srfD* mutant showed growth on the CD+PT+UU and CD+PT+UU+5-FOA plates, but not on the CD+PT plate without uridine and uracil; the *cpaA-R*::ptrA*-OE srfD-9 was identified as a correct mutant. CD: Czapek-Dox medium; PT: pyrithiamine; UU: uridine and uracil; 5-FOA: 5-fluoroorotic acid. c. agarose gel electrophoresis of *cpaA-R*::ptrA*-OE srfD mutants, further confirmed the genotype of *cpaA-R*::ptrA*-OE srfD -9, M: DNA marker.

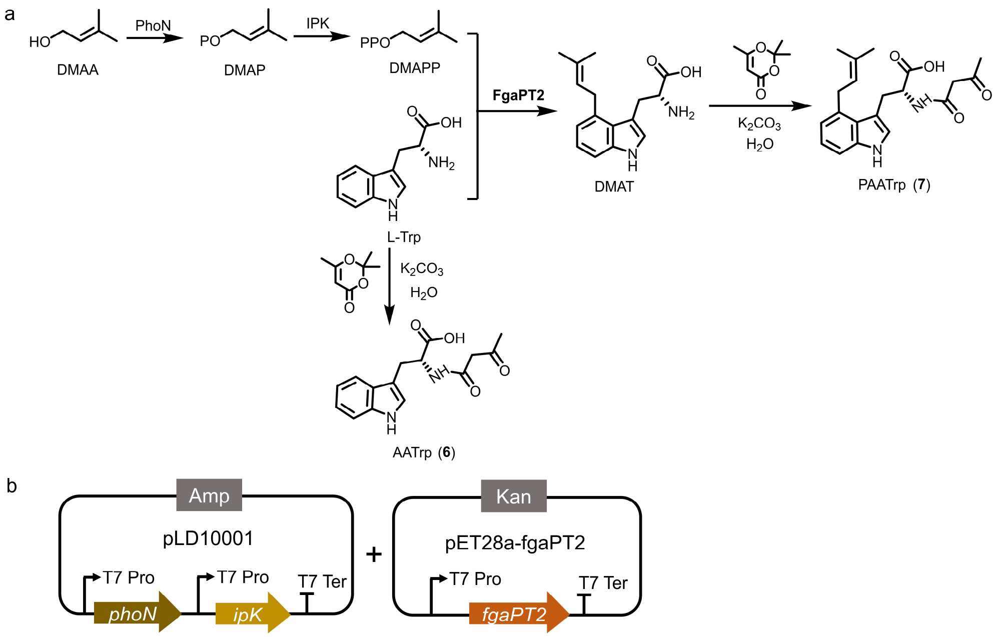

Figure S5. Synthesis of AATrp and PAATrp by whole cell biotransformation and chemical reactions (a). The plasmids used for biosynthesis of DMAPP (dimethylallyl diphosphate) (pLD10001) [34]and prenylation of L-tryptophan (pET28a-fgaPT2) in *E. coli* Rosetta (DE3) (b).

Table S5. NMR assignments for β-CPA, α-CPA and PAATrp (^{1}H, 600 MHz, ^{13}C 150 MHz).

	**β-CPA**		**α-CPA**		**PAATrp**	
**No.**	**^{1}H**	**^{13}C**	**^{1}H**	**^{13}C**	**^{1}H**	**^{13}C**
1			8.16 (1H br s)			176
2	7.03 (1H, m)	123.1	7.17 (1H s)	123.0	4.72 (1H, dd， *J* = 9.6, 4.4 Hz)	55.9
3		111.7		110.0	3.22 (1H, dd, *J* = 15.1, 9.5 Hz), 3.57 (1H, dd, *J* = 15.1, 4.6 Hz)	30.8
4	3.67 (2H, m), 2.85 (2H, m)	30.2	3.67 (1H dd, *J* =11.0, 5.9)	26.5		111.8
5	4.08 (1H, dd, *J* = 11.1, 3.3 Hz)	63.6	4.18 (1H, d, *J* =11.1)	71.8		126.4
6		194.5				135.2
7		101.8		63.4	6.75 (1H, d, *J* = 7.1 Hz)	120.4
8		174.7		36.1	6.96 (1H, t, *J* = 7.6 Hz)	122.4
9				26.4	7.15 (1H, d, *J* = 8.1 Hz)	110.4
10		133.0		128.7		138.8
11	5.33 (1H, m)	123.4	2.64 (1H, m)	116.5	7.08 (1H, s)	124.5
12	3.75 (2H, m)	32.4	3.07 (2H, m)	120.8	3.76 (2H, t, *J* = 5.7 Hz)	33.4
13		134.8		108.7	5.32 (1H, m)	125.6
14	6.93 (1H, d, *J* = 7.2 Hz)	120.6	7.20 (1H, d, *J* =8.0)	133.4		132.7
15	7.14 (1H, t, *J* = 7.6 Hz)	122.9	7.14 (1H, m)	125.9	1.76 (3H, s)	18.2
16	7.24 (1H, m, *J* = 8.2 Hz)	109.6	6.92 (1H, d, *J* = 6.9)	195.2	1.74 (3H, s)	25.9
17		137.4		105.6		168.8
18		124.8		175.2	3.3 (2H)	49.6
19		185.6		184.5		204.7
20	2.49 (3H, s)	19.8	2.46 (3H, s)	24.3	2.08 (3H, s)	29.8
21	1.75 (3H, d, *J* = 1.04 Hz)	18.3	1.65 (3H, s)	19.71		
22	1.77 (3H, s)	25.8	1.69 (3H, s)	19.71		

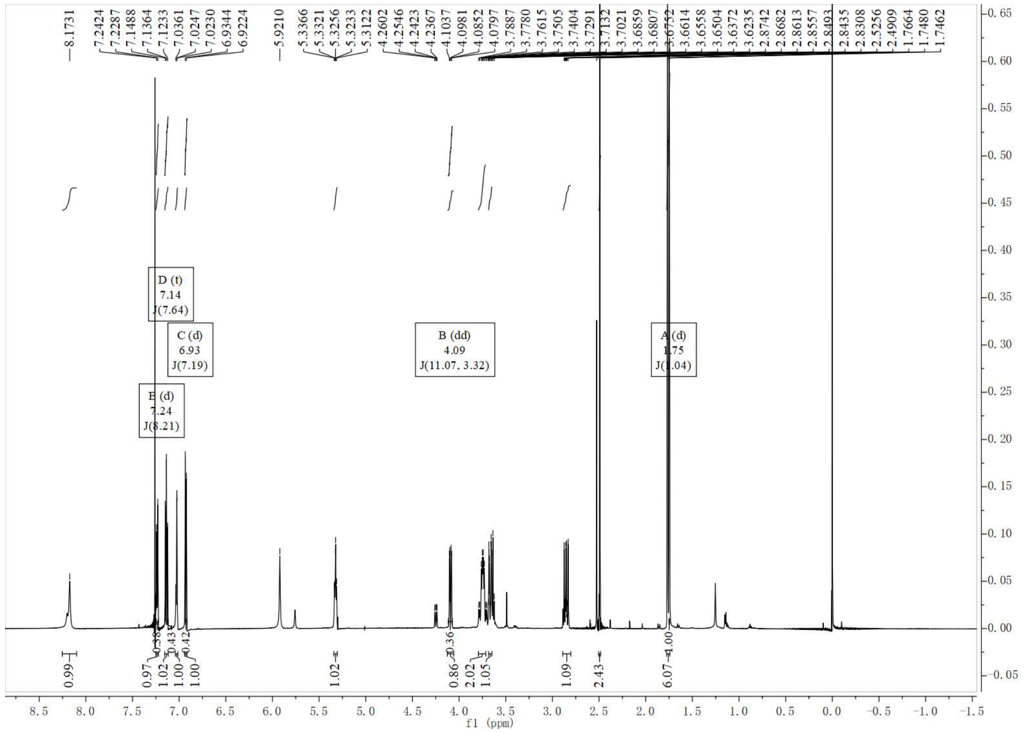

Figure S6. ^{1}H NMR spectrum of compound **2** (β-CPA) (600 MHz, $CDCl_3$).

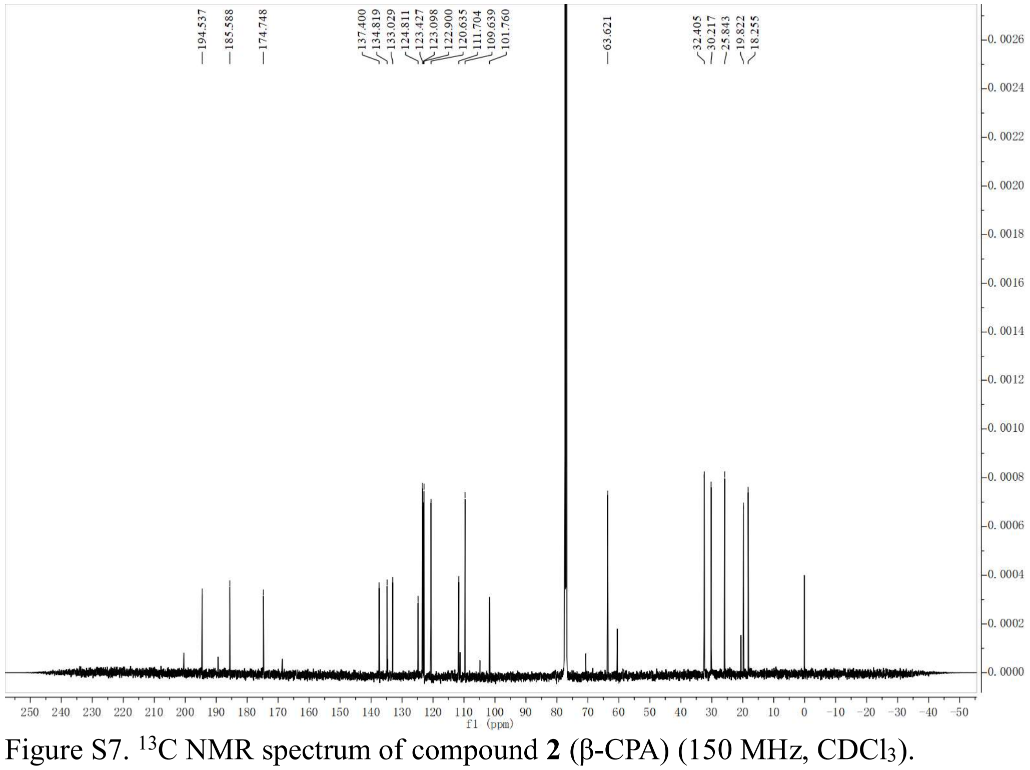

Figure S7. ^{13}C NMR spectrum of compound **2** (β-CPA) (150 MHz, $CDCl_3$).

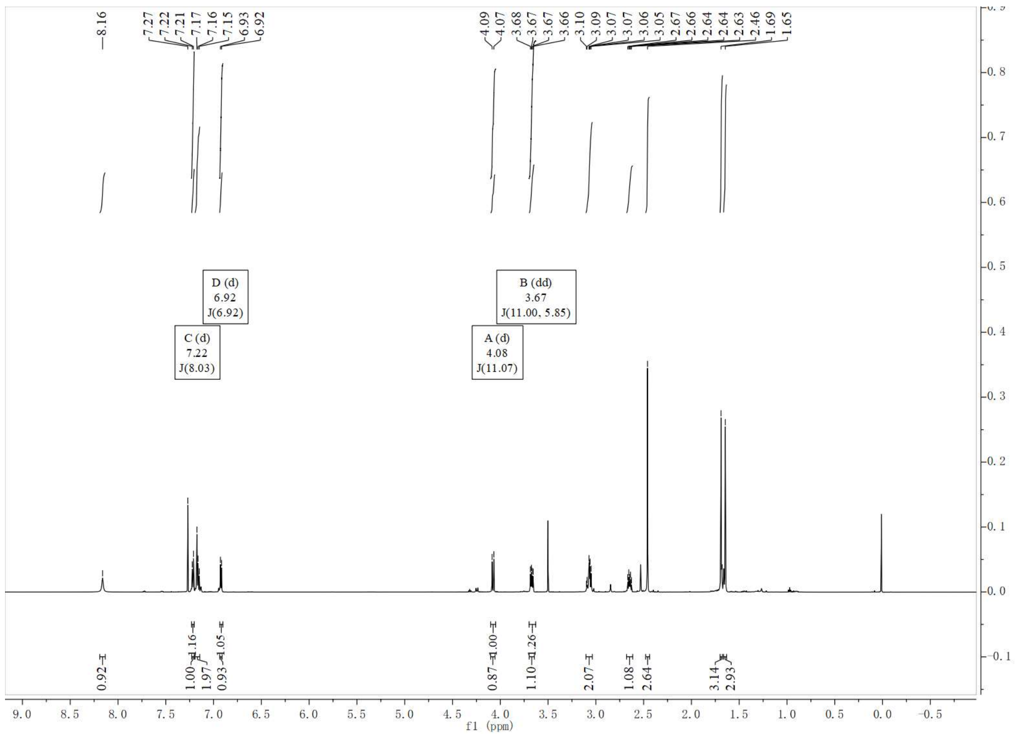

Figure S8. ^{1}H NMR spectrum of compound **3** (α-CPA) (600 MHz, $CDCl_3$).

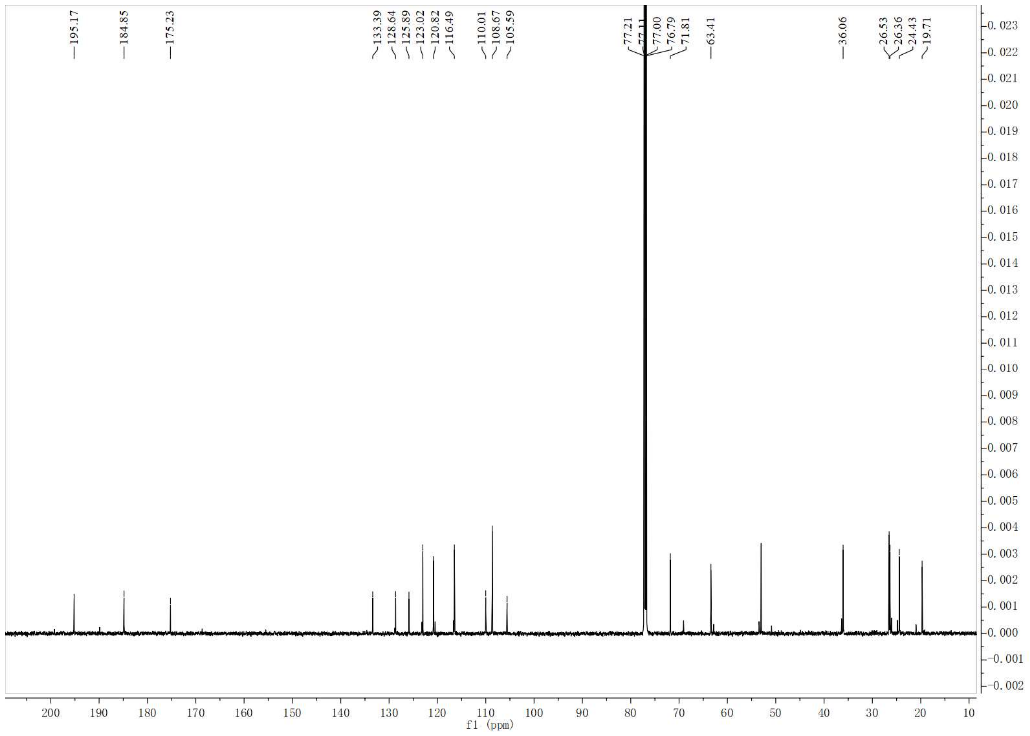

Figure S9. ^{13}C NMR spectrum of compound **3** (α-CPA) (150 MHz, $CDCl_3$).

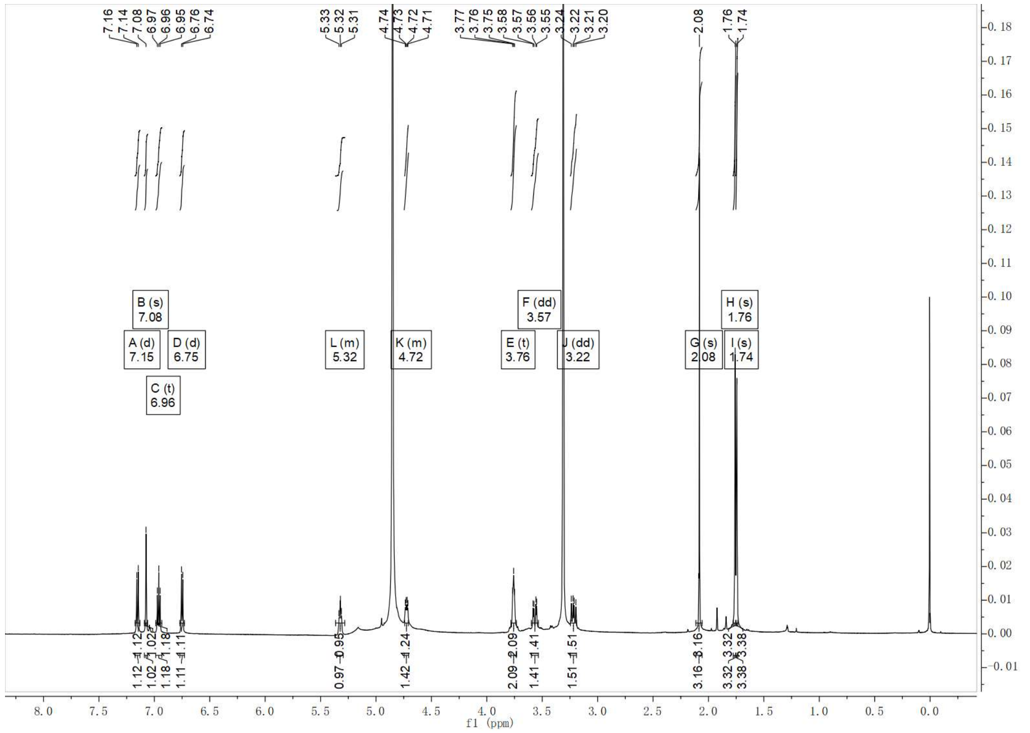

Figure S10. ^{1}H NMR spectrum of compound **7** (PAATrp) (600 MHz, $CDCl_3$).

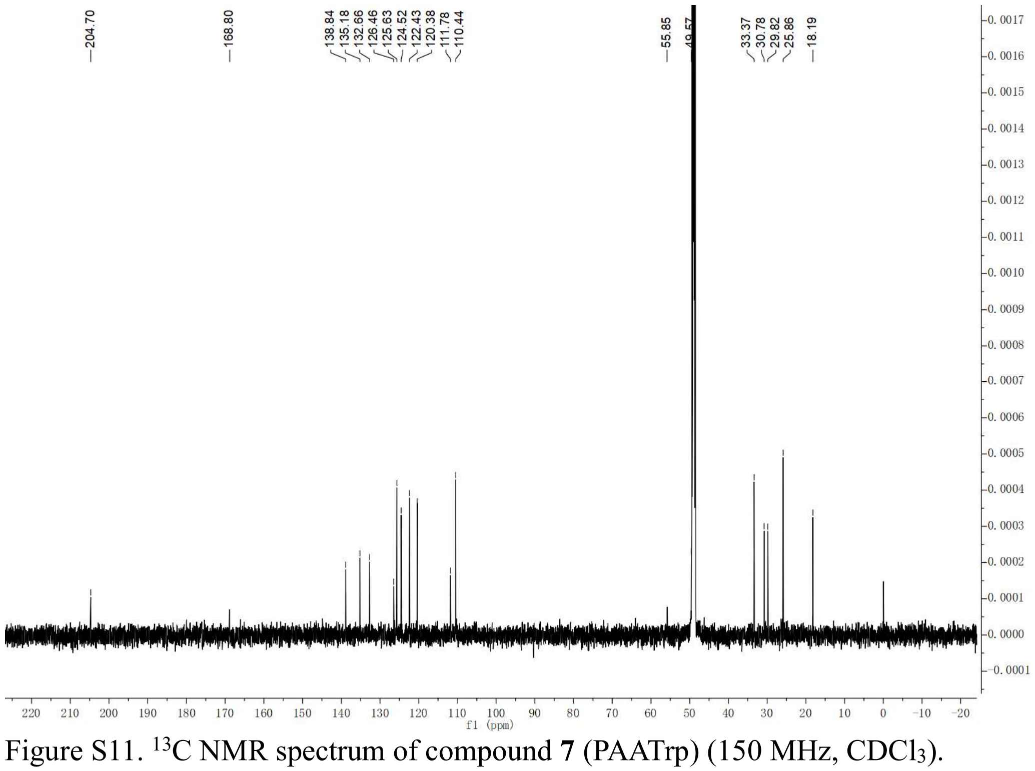

Figure S11. ^{13}C NMR spectrum of compound **7** (PAATrp) (150 MHz, $CDCl_3$).

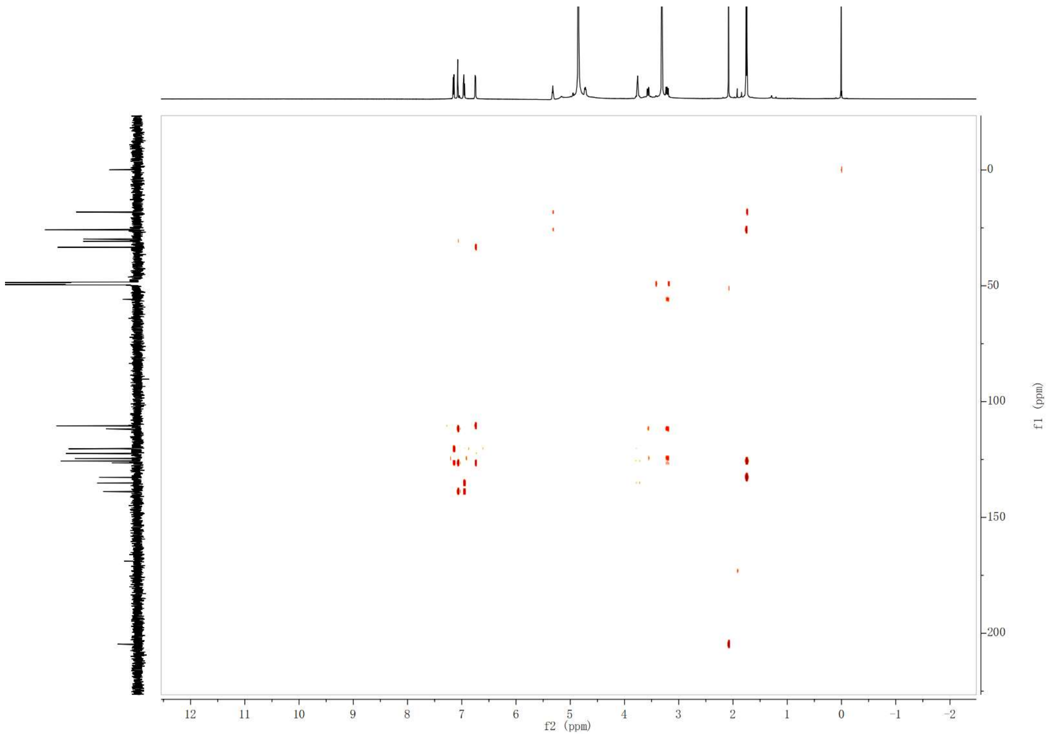

Figure S12. HMBC spectrum of compound **7** (PAATrp) (CD_3OD).

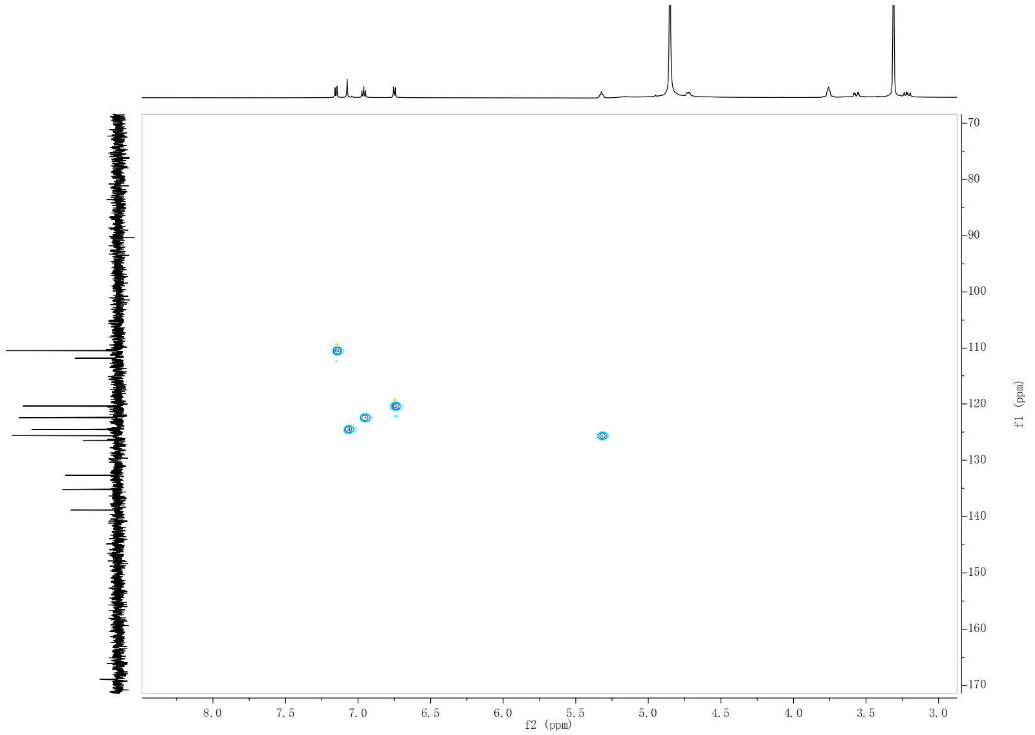

Figure S13. HSQC spectrum of compound **7** (PAATrp) (CD_3OD).

>acvA-TE

CCGATCTTGTTCCTATTGCCGCCAGGGGAGGGAGGGGCAGAAAGCTACTTTAACAATATCG
TCAAGCACTTGCCCACGACTAATATGGTCGTCTTTAACAATTACTACCTTCACTCCAAGAGT
CTGAACACGTTTGAAAAGCTAGCTGAGATGTATTTGGGGCACATCCGTCAGATCCAGCCAG
ACGGGCCTTACCATTTCATCGGATGGAGTTTTGGAGGAACAATCGCGATGGAAATATCGCGA
CAGCTCGTGGGGCTAGGTTCAACGATTGGTCTTTTAGGTATCATTGACACGTATTTCAACGT
GCCTGGAGCAACGCGGGCAATTGGCCTCGGTGATACTGAGGTCTTGGATCCCATTCATCATA
TATCCCAACCAGAACCAGCCGATTTCCAGTGCCTCCCAGCCAGCACAGACTACATCATTTTA
TTCAAAGCTACTAGGGTGAACGACAAGTTTCAGTCTGAAAACCAGAGGCGTCTGTACGAG
TACTACGACAAAACATTGCTTAATGATCTCGACTGGTTACTCCCTGGTGCTTCAAACATTCAT
CTAGTCCGCCTTGAGGAGGATACTCACTTCTCCTGGGCGACCAATCCACGCCAAATCGCCC
ACGTTTGTTCAACAATCGAGAAATTTCTC

>fgaPT2

TCAGTGGAGACCGGAATAATATACCCCTGTCTTTGACAGATCTGGAGGTAAAGCTGTGGGTT
GACAGGCCGCATCCTGACACTTGACCTTTGATTCGGATAAGTTTGCAACTGCCCAGTCCCCT
GTCTCGAAGGATTGAAGATAGACACTCAGATAAGGGGTACGGTCCCTGTAGGAGAAGGATA
TGTAGGCGTGGAGGTAGTTAAGTTTGTCATGATCCGCATGGGGGTAGTACGACTTCAAAGTA
GTTTCGTAGGTGCGGGCCATTTCACTCCAACCCCGGCGCTCGAAGAACGTCGTCAGGGCAT
CCGCCACCGCCATGTCGTTCATGCCGAAGGTTGTGAAATATACTTGCGGCTCTGGGACCGG
GTCATTCTGGTGCAGGGTGAAATTGGCCATAAGCGGCAGCCTCTCGTCTGGGATAACCCCG
AGAGGCAGATACGGCGCCGGATAGGACTTCAATCCCGGCGACAGTTGGATCAGATCCCAAA
GCTCACGCACCAGAGAGAGCCCCTCTAAAGTGGAAGCGTCTCGGCGCCGTCCGCCCAGAG
TCCACAGGTCCTCCATGGCTTCTAGTGAAACCATCTGCTCCAGCAGGTAGATCTTGATTCTC
GACTTGGCAGGACTGGTCAGATCACAGGACACTAGGCGGGGACTGGCAGTGCTCTTGGAA
CCGCGTGATCGGATGTATTCCTCCAGCATGTTGAGTGGGGGCAAGATTCGGGGCTCCCTCA
CTGCCAGCCGGCGGACTGAGCCAAAGACCAACTCATGAATTGTCTTGCCGGTGACGACAG
CTTTGAGAGCCGGGTATATGTACGTCTTAAGTGCAAAGCGGCCATCCTTCAGATCGAGCGCG
AGCTTGTTCTGCGTCCTGATAGTGCCGCCCACGAGGCGATCATTATGAGCCAGAAAAGCAG
ATTCTTCACTGTTGAGGGTGAGATCGTGCTTGAAGTGGCGGAACCACTCCAGATCAATGCT
CTTCTCCAGTGGAAGCAGGTGCTGCAGGCTCTCCCAGATGGCGTGCGTATTGAATGGGTCT
TTATCTGTTCCGGTATGTTGATTGATCGGCTCGAATGTGTATCTCACTATTGAATTAGAGCAAT
TTAGGCTCAATTCGAACGGAGTGCCGTATCGAGTGAGGATGCTCAACCAGCGGGGGGCGCT
GTTGGTCGGATAGCATCCGAGACTGGGAATTACGCACTCCTTATAGGTGATGAGGTATTGAT
ACTGACAAGGTGTGGTGTAGTTGGCAGTTTCCAGCATTTTTGCAAACATCGGGGCAGTGCT
GTGCCACCACAGCTTCTGATCTTCATTATCGAATCTAAAGGCGCGACTAAGAACTCGATAGG
CCTCCGCACTGGAGGCATTGGCTGCCTTCAT
